# Involvement of the long noncoding RNA NEAT1 in carcinogenesis

**DOI:** 10.1002/1878-0261.12404

**Published:** 2018-12-03

**Authors:** Christiane Klec, Felix Prinz, Martin Pichler

**Affiliations:** ^1^ Division of Oncology Department of Internal Medicine Medical University of Graz (MUG) Austria; ^2^ Research Unit for Non‐coding RNAs and Genome Editing Medical University of Graz (MUG) Austria; ^3^ Department of Experimental Therapeutics The University of Texas MD Anderson Cancer Center Houston TX USA

**Keywords:** cancer, ceRNA, lncRNA, miRNA, NEAT1

## Abstract

Altered expression levels of the long noncoding RNA (lncRNA) nuclear‐enriched abundant transcript 1 (NEAT1) have been reported in different types of cancer. More than half of the NEAT1 studies in cancer have been published within the last 2 years. In this review, we discuss very recent developments and insights into NEAT1 contribution to carcinogenesis. Summarizing the literature, it becomes obvious that NEAT1 is a lncRNA highly de‐/upregulated in a variety of cancer entities, in which it primarily acts as a competing endogenous RNA (ceRNA) which sponges tumor‐suppressive microRNA (miRNA). The sponged miRNA lose their ability to degrade, silence, or hamper translation of their downstream—mostly oncogenic—target transcripts, ultimately promoting carcinogenesis. This role of NEAT1 function in tumorigenesis suggests it may be a prognostic biomarker as well as potential therapeutic target, pending the completion of further studies into the underlying mechanisms.

**Abbreviations**
ceRNA: competing endogenous RNA
EMT: epithelial–mesenchymal transition
IRAlu: inverted repeat Alu elements
lncRNA: long noncoding RNA
miRNA/miR: microRNA
ncRNA: noncoding RNA
NEAT1: nuclear‐enriched abundant transcript 1
p54nrb/NoNO: 54 kDa nuclear RNA‐ and DNA‐binding protein/Non‐POU domain‐containing octamer‐binding protein
PSF/SFPQ: PTB‐associated splicing factor/splicing factor proline glutamine rich
SIM: structured illumination microscopy

## 1 Introduction

Since results of genome‐wide studies have shown that approximately 70% of the human genome is transcribed into RNA but < 2% is protein‐coding (Consortium, 2012), noncoding RNA (ncRNA) have come more into focus of research (Ling *et al*., 2015; Nana‐Sinkam and Croce, 2011). Technical progress in sequencing technologies led to the discovery of the huge family of ncRNA. In the last 2 years, extensive research was done in this field and long noncoding RNA (lncRNA), typically > 200 nucleotides in length, have on the one hand been found to be implicated in a variety of biological processes such as gene expression, subcellular architecture, and stabilization of protein complexes (Gutschner *et al*., 2018; Kung *et al*., 2013; Reicher *et al*., 2018; Smolle *et al*., 2017). On the other hand, studies with lncRNA proved their involvement in physiology and pathophysiology (Chen *et al*., 2014; Del Vecchio *et al*., 2018; Schanza *et al*., 2017; Smolle and Pichler, 2018). Besides mRNA, pseudogenes, and circular RNA, also lncRNA often function as competing endogenous RNA (ceRNA) in a variety of pathologies (Cesana *et al*., 2011; Hansen *et al*., 2013; Memczak *et al*., 2013). ceRNA are transcripts able to regulate each other at the post‐transcriptional level by competing for shared microRNA (miRNA), or more precisely, they act as natural molecular sponge for miRNA (Qi *et al*., 2015). Consequently, the sponged miRNA lose their ability to degrade, silence, or hamper the translation of their downstream target transcripts (Bartel, 2009). The mechanism underlying the ceRNA hypothesis was first described in 2007 by Franco‐Zorrilla *et al*. where they reported that the lncRNA induced by phosphate starvation 1 (IPS1) regulates PHO2 protein levels in plants by limiting the availability of miR‐399 and therefore inhibiting the repressive function of miR‐399 on PHO2 mRNA (Franco‐Zorrilla *et al*., 2007). In the same year, Ebert *et al*. reported on a new technique controlling endogenous miRNA levels by expressing competitive inhibitors containing multiple, tandem binding sites for specific miRNA in cell systems resulting in a sponging of these miRNA. This group for the first time introduced the term ‘miRNA sponge’ (Ebert *et al*., 2007). These early studies report on the principle of miRNA target regulation by competitively sequestering miRNA affecting the activity of their targets. Based on these findings, Salmena *et al*. proposed the hypothesis of ‘ceRNA’ in 2011 stating that RNA transcripts (i.e., messenger RNA, transcribed pseudogenes, or lncRNA) containing microRNA‐response elements (MREs) are able to exert the function as ceRNA de‐repressing the activity of other RNA with similar MREs by competing for the same miRNA in the available miRNA pool (Salmena *et al*., 2011).

The subject of lncRNA exerting ceRNA function contributing to carcinogenesis is currently under extensive investigation, and many lncRNA have been demonstrated to be molecular sponges for miRNA in several cancer entities, for example, AFAP1‐AS1 in nasopharyngeal carcinoma (Lian *et al*., 2018) and pancreatic cancer (Chen *et al*., 2018), FLVCR1‐AS1 in lung cancer (Gao *et al*., 2018), or TP73‐AS1 in gastric cancer (Ding *et al*., 2018) to name just a few. Thomson *et al*. published a detailed review critically discussing the evidence and controversy of miRNA sponges (Thomson and Dinger, 2016).

This review will give an overview of the most recent findings on the lncRNA NEAT1, especially focusing on the consequences of its deregulation and its function as ceRNA in the development of cancer.

## 2 NEAT1—history, structure, function

Nuclear‐enriched abundant transcript 1 has been discovered in 2007 by Hutchinson *et al*. as being a lncRNA enriched in the nucleus localized within paraspeckles (Hutchinson *et al*., 2007) and was found to be essentially needed for paraspeckle integrity (Clemson *et al*., 2009). Two variants of this lncRNA exist; that is, NEAT1_1 (3.7 kb) and NEAT1_2 (23 kb) encoded by the NEAT1 gene (Sasaki *et al*., 2009) and transcribed from the multiple endocrine neoplasia locus in human chromosome 11qA (Guru *et al*., 1997). Paraspeckles are nuclear complexes consisting of proteins, that is, PTB‐associated splicing factor/splicing factor proline glutamine rich (PSF/SFPQ; Prasanth *et al*., 2005), 54 kDa nuclear RNA‐ and DNA‐binding protein/Non‐POU domain‐containing octamer‐binding protein (p54NRB/NONO) and paraspeckle component 1 (PSPC1; Fox *et al*., 2002), and the lncRNA NEAT1. Paraspeckles are responsible for regulating gene expression by retaining A‐I‐edited mRNA in the nucleus, whereas unedited RNA are transported into the cytoplasm (Zhang and Carmichael, 2001).

### 2.1 NEAT1 structure

A combination of immunofluorescence and fluorescent *in situ* hybridization (FISH) experiments revealed an intensive colocalization of NEAT1 lncRNA with paraspeckle proteins p54nrb/NONO and PSP1 (Clemson *et al*., 2009), which have earlier been shown to form heterodimers within paraspeckles (Fox *et al*., 2005). The interaction between NEAT1 and p54nrb/NONO happens through three protein interaction sites localized near the 5′ and 3′ ends of NEAT1 (Murthy and Rangarajan, 2010). Further elegant studies combining FISH (RNA detection) and structured illumination microscopy (SIM) allowed the simultaneous detection of the RNA and protein components of paraspeckles, which was not possible with standard fluorescent microscopy due to resolution limits (Mito *et al*., 2016). Detailed analyses with the SIM technique uncovered the structural organization of NEAT1 within paraspeckles; that is, the paraspeckle components are arranged in a core‐shell spheroidal structure, whereas the 5′ and 3′ ends of NEAT1 are located at the periphery of the paraspeckles and the central sequence of NEAT1 is localized within the core (West *et al*., 2016). In contrast to the long NEAT1 isoform, the short isoform is not a major component of paraspeckles but rather localizes to so‐called microspeckles and therefore probably exerting other regulatory functions (Li *et al*., 2017b). CRISPR/Cas9 deletion experiments provided more explicit information on the functional domains of NEAT1‐dependent paraspeckle organization. Yamazaki *et al*. provided details of two prerequisites essential for paraspeckle formation, that is, (a) the middle domain of NEAT1 which is sufficient for the formation and (b) the binding of p54nrb/NONO to this middle domain via its NOPS (NONA/paraspeckle) dimerization domain. Due to its architectural role in paraspeckle structure, authors defined NEAT1 as being an architectural RNA (Yamazaki *et al*., 2018b). By selectively overexpressing NEAT1 with the CRISPR/Cas9 synergistic activation mediator (SAM) system, the same group was able to induce intact paraspeckles with ordered core‐shell structure. This approach allows more detailed studies in the future on the functional role of NEAT1 (Yamazaki *et al*., 2018a).

### 2.2 NEAT1 function

As mentioned above, NEAT1 was first discovered with expression array experiments in 2007 in parallel with NEAT2 also known as metastasis‐associated lung adenocarcinoma transcript 1 (MALAT1) as approximately 4‐kb‐long unspliced, polyadenylated nuclear‐restricted noncoding transcript which is broadly expressed with high abundance in ovary, prostate, colon, and pancreas. Already in early studies, the existence of a second > 17‐kb‐long isoform with lower expression rate was suggested. Authors proposed a localization of NEAT1 within paraspeckles which are described as structures on the edges of nuclear speckles (Hutchinson *et al*., 2007). The Pol II‐transcribed NEAT1 was shown to be essential for paraspeckle integrity since a depletion of NEAT1 eradicates them. Overexpression of NEAT1 increases the amount of paraspeckles within the cells, while PSP1 overexpression does not indicating NEAT1 as the bottleneck for paraspeckle formation. Physiologic implications of NEAT1 have been investigated by several groups where they show that the short isoform of NEAT1 is broadly expressed in a wide range of tissues, whereas the long isoform is expressed in a subpopulation of cells in adult mice with highest abundance in stomach and intestine. Accordingly, intense paraspeckle formation, which crucially depends on the long isoform, is observed in cellular subpopulations of living mice. Since NEAT1 knockout mice are viable and fertile and do not show an apparent phenotype, authors propose that paraspeckles are nonessential subpopulation‐specific nuclear bodies (Nakagawa *et al*., 2011). NEAT1 is essential for corpus luteum formation and defines fertility in a subpopulation of mice (Nakagawa *et al*., 2014), is fundamental for mammary gland development and lactation capacity in mice (Standaert *et al*., 2014), and modulates neuronal excitability in humans (Barry *et al*., 2017). The functional role of NEAT1 in the cellular context is not fully uncovered yet, but early studies show an involvement in the sequestration of paraspeckle proteins of the drosophila behavior human splicing (DBHS) family, that is, PSP1 and p54nrb/NONO which are implicated in A‐I editing of mRNA. Although NEAT1 itself is not A‐I edited, it is nevertheless retained in the nucleus; therefore, authors propose an architectural role of this lncRNA (Clemson *et al*., 2009). One component of the PSP1‐p54nrb/NONO‐NEAT1 complex namely p54nrb/NONO is involved in the retention of A‐I‐edited mRNA by dsRNA‐dependent adenosine deaminases (ADARs) preventing the nuclear export of these mRNA containing inverted repeated Alu (IRAlu) elements within the 3′UTR (Chen *et al*., 2008). In human embryonic stem cells (hESC), mRNA containing IRAlu elements are not as one would expect retained in the nucleus except after they have differentiated. The explanation for this phenomenon is the absence of paraspeckles in undifferentiated hESC because of lacking NEAT1 expression. Upon differentiation, NEAT1 expression is started leading to paraspeckle formation enabling nuclear retention of mRNA and therefore representing an interesting functional role of NEAT1 in the process of differentiation (Chen and Carmichael, 2009). Paraspeckle‐driven nuclear retention is controlled by coactivator‐associated arginine methyltransferase 1 (CARM1) on two levels. On the one hand, CARM1 methylates p54nrb/NONO disabling its binding to mRNA containing IRAlu elements and on the other hand suppresses NEAT1 transcription reducing the amount of paraspeckles. Both events lead to less nuclear retention of mRNA and provide another regulatory mechanism in which NEAT1 is involved (Hu *et al*., 2015). Another functional involvement of NEAT1 was shown by Imamura *et al*. and Hirose *et al*., that is, transcriptional regulation due to NEAT1‐dependent PSF/SFPQ sequestration to paraspeckles. PSF/SFPQ is a repressor of interleukin 8 (IL‐8) transcription. Upon viral infection, NEAT1 expression is induced leading to a NEAT1‐dependent relocation of PSF/SFPQ from the IL‐8 promotor to paraspeckles activating IL‐8 transcription and subsequent stimulating immune response (Imamura *et al*., 2014). Another example for transcriptional regulation executed by NEAT1‐dependent PSF/SFPQ relocation is the control of RNA‐specific adenosine deaminase B2 (ADARB2) transcription which requires PSF/SFPQ. Upon cellular stress induced with proteasome inhibition, NEAT1 expression is upregulated subsequently sequestering PSF/SFPQ to paraspeckles reversing its binding to the ADARB2 promoter and subsequently leading to reduced ADARB2 transcription (Hirose *et al*., 2014).

## 3 NEAT1 in cancer biology

In this section, the involvement of NEAT1 in cancer biology will be discussed. Research concerning this topic before 2017 has been very well reviewed by Lo *et al*. (2016a) and Yu *et al*. (2017). The next part will mainly focus on knowledge gained within the last 2 years providing detailed information about the most commonly studied cancer types, that is, nonsmall lung cancer, breast cancer, hepatocellular carcinoma, ovarian cancer, and prostate cancer. Meta‐analyses clearly show that the lncRNA NEAT1 is upregulated in various cancer entities resulting in an unfavorable prognosis as well as a poor overall survival and, thus, these studies conclude that NEAT1 could be a suitable prognostic biomarker candidate for clinicopathological features in cancer pathology (Chen *et al*., 2017b; Yang *et al*., 2017a; Zhang *et al*., 2017c). A recent pan‐cancer study predicts that NEAT1 is the lncRNA which has the most cancer gene targets closely followed by the lncRNA MALAT1, LINC00969, and OIP5‐AS1 (Chiu *et al*., 2018). Contradictory findings show that NEAT1 is downregulated in some cancers (e.g., acute promyelocytic leukemia (Zeng *et al*., 2014)) and is a downstream target of the tumor suppressor p53, thus proposing that NEAT1 works as a potential tumor suppressor (Adriaens *et al*., 2016; Idogawa *et al*., 2017; Mello *et al*., 2017).

### 3.1 Breast cancer

The first evidence that NEAT1 plays a crucial role in breast cancer biology was delivered by Choudhry *et al*. when they showed that NEAT1 is a direct target of hypoxia‐inducible factor 2 (HIF‐2), which is known to be activated in cancer (Lofstedt *et al*., 2007). HIF‐2 was shown to transcriptionally regulate NEAT1. Under hypoxia—which leads to HIF‐2 activation—NEAT1 is upregulated and consequently paraspeckle formation is induced, resulting in a nuclear retention of F11R, a factor which was earlier shown to be retained in the nucleus under hypoxic conditions (Ben‐Zvi *et al*., 2013). Choudhry *et al*. could prove that F11R retention depends on hypoxia‐induced NEAT1 upregulation. Increased cellular proliferation and clonogenic survival as well as decreased apoptosis of breast cancer cells are the outcome of hypoxia‐induced NEAT1 upregulation (Choudhry *et al*., 2015).

Since 2016, a lot of research was done on NEAT1 contribution to breast cancer progression and several studies have shown a connection of this lncRNA with different miRNA. MiRNA are small endogenous RNA that regulate gene expression pattern through direct interaction with larger messenger RNA. Ke *et al*. found a correlation between NEAT1 and mir‐548ar‐3p with fused in sarcoma/translocated in liposarcoma (FUS/TLS). Knockdown of NEAT1 leads to reduced cellular growth and increased apoptosis of breast cancer cell lines. FUS and NEAT1 directly interact, and also, a knockdown of FUS resulted in increased apoptosis. miR‐548 was shown to regulate NEAT1 expression since overexpression of miR‐548 leads to decreased NEAT1 expression, ultimately inducing apoptosis (Ke *et al*., 2016).

Nuclear‐enriched abundant transcript 1 is also a target of breast cancer susceptibility gene 1 (BRCA1), which is commonly mutated in hereditary cases of breast cancer (Miki *et al*., 1994). BRCA1 deficiency increases NEAT1 expression and boosts tumorigenicity *in vitro* and *in vivo*. NEAT1 negatively regulates miR‐129‐5p by increasing DNA methylation of CpG islands in the miR‐129‐5p gene. Reduced miR‐129‐5p levels result in augmented WNT4 expression activating oncogenic WNT signaling. The authors concluded that the BRCA1/NEAT1/miR‐129‐5p signaling axis contributes to breast cancer tumorigenesis (Lo *et al*., 2016b).

Recent studies showed that NEAT1 is upregulated in breast cancer cell lines as well as in patient tumor tissue and that this upregulation is associated on the one hand with increased cell growth and proliferation, invasion, promoted epithelial–mesenchymal transition (EMT), reduced apoptosis in breast cancer cell lines and on the other hand with an unfavorable prognosis and overall patient survival, increased tumor size, lymph node metastasis, and cancer aggressiveness in patients (Li *et al*., 2017c,d; Qian *et al*., 2017; Zhang *et al*., 2017b; Zhao *et al*., 2017). Upregulated NEAT1 expression increases breast cancer cell growth by targeting miR‐101, a miRNA which was shown to negatively correlate with NEAT1 expression levels. miR‐101 targets enhancer of zeste homolog 2 (EZH2), a marker for aggressive breast cancer (Kleer *et al*., 2003), and an upregulation of miR‐101 results in decreased EZH2 levels. Thus, authors propose that NEAT1 knockdown might repress cancer cell growth via miR‐101‐dependent EZH2 regulation (Qian *et al*., 2017). As mentioned above, upregulation of NEAT1 promotes EMT, which triggers 5‐fluorouracil (5‐FU) resistance (Wu *et al*., 2016b). Li *et al*. showed that NEAT1‐induced EMT as well as chemo‐resistance of breast cancer cells is regulated via miR‐211/HMGA2 axis. miR‐211—known for inhibiting cancer cell migration and invasion (Chen *et al*., 2017a)—is repressed by upregulated NEAT1 and consequently leading to an upregulation of the EMT inducer high‐mobility group AT‐hook 2 (HMGA2; Wu *et al*., 2016a). Authors conclude that NEAT1 sponges miR‐211 and thus inhibits its repressor function on HMGA2 (Li *et al*., 2017d).

Nuclear‐enriched abundant transcript 1 was shown to be responsible for the interaction between forkhead/winged helix transcription factor 3 (FOXN3) and paired amphipathic helix protein 3 (SIN3A) in hormonally dependent breast cancer, forming a complex repressing genes such as trans‐acting T cell‐specific transcription factor 3 (GATA3), which is under normal conditions an EMT repressor (Yan *et al*., 2010). The NEAT1/FOXN3/SIN3A axis is promoting EMT and is responsible for dissemination and metastasis formation *in vivo* (Li *et al*., 2017c).

Jiang *et al*. detected increased NEAT1 levels in diverse breast cancer cell lines compared to MCF‐10A cells (normal mammary epithelial cells). The upregulation of NEAT1 negatively correlated with miR‐448 expression, which is a known inhibitor of cancer cell growth (Ma *et al*., 2018). NEAT‐1‐induced sponging of miR‐448 removes the inhibitory effect of this miRNA on ZEB1 (Jiang *et al*., 2018b), thus upregulating this transcription factor responsible for cancer progression by promoting EMT (Graham *et al*., 2010).

The reason for all these involved miRNA and different regulatory pathways might be explained in the excellent recent work of Zhou *et al*. They showed the implication on lncRNA in the four subtypes of breast cancer, that is, (a) basal‐like, (b) HER2+, (c) luminal A, and (d) luminal B. NEAT1 was one of three lncRNA—besides OPI5‐AS1 and AC008124.1—involved in all four subtypes. These lncRNA exert specific roles in the ceRNA network by competing with diverse miRNA, and an aberrant expression could disrupt the network structure. NEAT1 was shown to compete with different RNA within the four breast cancer subtypes and thus exerting diverse regulatory functions on cell activities. For example, in the basal like type NEAT1 competes with TGFB1 influencing vasculogenesis which is the basis for tumorigenesis. In the HER2+ type, NEAT1 competes with LDHA regulating glycolytic processes within cancer cells (Zhou *et al*., 2018).

### 3.2 Nonsmall cell lung cancer

In the last 2 years, several studies indicating the involvement of NEAT1 in nonsmall cell lung cancer (NSCLC) were published. All of these studies have in common that they show an upregulation of NEAT1 in cancer tissue as well as in cell lines and that this increase in NEAT1 levels was associated with more lymph node metastasis, higher TNM grades, and a poor overall survival in patients as well as increased proliferation, invasion, and migration *in vitro* (Jen *et al*., 2017; Li *et al*., 2018a; Sun *et al*., 2017; Wu *et al*., 2017; Zhang *et al*., 2017a). Also, in NSCLC NEAT1 is promoting tumorigenesis by regulating diverse molecular pathways. Sun *et al*. could associate NEAT1 with Wnt/β‐catenin signaling (Sun *et al*., 2017). The Wnt/β‐catenin axis contribution to breast cancer and nonsmall lung cancer has been shown before, that is, an activation of Wnt/β‐catenin signaling is promoting tumorigenesis (Fu *et al*., 2016; Xiao *et al*., 2016). Knockdown of NEAT1 leads to an inhibition of Wnt/β‐catenin consequently resulting in reduced proliferation, invasion, and aggressiveness of NSCLC (Jiang *et al*., 2018a; Sun *et al*., 2017). The expression of the transcription factor octamer‐binding transcription factor 4 (Oct4), which has been shown to be upregulated as a stem cell factor in several cancer entities (Villodre *et al*., 2016), positively correlates with NEAT1 expression in NSCLC patients. Jen *et al*. propose that Oct4 controls NEAT1 transcription since Oct4 overexpression leads to an increased NEAT1 expression. Rescue experiments in Oct4‐silenced cells show that NEAT1 overexpression is re‐establishing cancer cell proliferation (Jen *et al*., 2017). Recently, three groups created a connection between NEAT1 and miRNA in NSCLC by showing NEAT1 to be a ceRNA for these miRNA. (a) NEAT1 acts as a sponge for miR‐181a‐5p—high NEAT1 levels inversely correlate with miR‐181a‐5p levels—leading to an upregulation of high‐mobility group box 2 (HMGB2; Li *et al*., 2018a)—a protein known for being upregulated and being the driver for tumorigenesis in several cancer types (Fu *et al*., 2018; Kwon *et al*., 2010). (b) NEAT1 promotes E2F3 expression by competitively binding miR‐377‐3p. The higher E2F3 expression of NEAT1‐induced sponging of miR‐377‐3p levels results in increased proliferation by cell cycle regulation (Zhang *et al*., 2017a). (c) The group of Wu *et al*. investigated the impact of the NEAT1/miR‐98‐5p/MAPK6 axis on NSCLC. NEAT1 again is competitively sponging miR‐98‐5p, where high miR‐98‐5p levels are inhibiting cancer cell growth, migration, and invasion on the one hand and reducing mitogen‐activated protein kinase 6 (MAPK6) levels on the other hand. The authors showed that NEAT1 is sponging miR‐98‐5p which in turn upregulates MAPK6. MAPK6 has been found to be upregulated in breast and gastric cancer being responsible for promoted tumorigenesis in these cancer types (Evtimova *et al*., 2001; Liang *et al*., 2005).

### 3.3 Hepatocellular carcinoma

Recent studies elucidating the role of NEAT1 in hepatocellular carcinoma could show an upregulation of this lncRNA in HCC patient tissue and HCC cell lines. A reduction of NEAT1 expression levels by knocking down the gene leads to decreased cell viability, cell growth, migration, invasion, and EMT *in vitro* as well as to reduced tumor size and metastasis *in* *vivo* (Fang *et al*., 2017; Fu *et al*., 2017; Tu *et al*., 2018; Wang *et al*., 2017c; Zhang *et al*., 2018). NEAT1 is crucially interplaying with miRNA also in hepatocellular carcinoma acting as a ceRNA and therefore reducing expression of these miRNA in the case of (a) miR‐129‐5p (Fang *et al*., 2017; Fu *et al*., 2017), (b) miR‐613 (Wang *et al*., 2017c), (c) miR‐485 (Zhang *et al*., 2018), and (d) miR‐139‐5p (Tu *et al*., 2018). Ad i., Liu *et al*. proved an involvement of miR‐129‐5p in HCC progression. miR‐129‐5p directly targets valosin‐containing protein (VCP) and IκB, both of which are known contributors to HCC progression. Overexpression of miR‐129‐5p decreased VCP and increased the NFκB inhibitor IκB expression resulting in augmented apoptosis and decreased migration of HCC cells (Liu *et al*., 2012). Fang *et al*. expanded this mechanism by showing that NEAT1‐induced sponging of miR‐129‐5p results in increased VCP and decreased IκB levels ultimately activating NFκB pathway promoting tumor progression (Fang *et al*., 2017). Ad ii., NEAT1 sponging of miR‐613 was discovered in 2017 by Wang Z. *et al*. One year earlier, the underlying mechanism of the tumor‐suppressive features of this miRNA was discovered by Wang W. *et al*. This group showed that downregulated miR‐613 leads to an increase of doublecortin‐like kinase 1 (DCLK1). DCLK1 is a microtubule‐associated protein and serves as tumor stem cell marker. This protein is often upregulated in solid tumors and is able to drive tumorigenesis (Wang *et al*., 2016). To summarize this, upregulated NEAT1 levels in HCC sponge miR‐613 ultimately leading to cancer progression due to upregulation of DCLK1. Ad iii., in case of miR‐485, authors show that this miRNA directly regulates expression levels of signal activator and transducer of transcription 3 (STAT3); that is, overexpression of miR‐485 results in decreased STAT3 expression and vice versa. Increased NEAT1 levels in HCC sponge miR‐485 and increased levels of STAT3 oncogene (Avalle *et al*., 2017) are the consequence (Zhang *et al*., 2018). Ad iv., for the interplay between NEAT1 with miR‐139‐5p, authors proved TGF‐β1—commonly upregulated in several cancer types regulating cancer progression and metastasis (Bierie and Moses, 2006)—being a downstream target of this miRNA which is upregulated by NEAT1‐induced sponging of miR‐139‐5p leading to promoted HCC proliferation and invasion (Tu *et al*., 2018). The recent work of Liu *et al*. provides information about a contribution of lipolysis in HCC. Adipose triglyceride lipase (ATGL) is upregulated in HCC and is associated with poor prognosis since increased ATGL levels lead to promoted HCC cell growth and colony formation. NEAT1 modulates ATGL expression leading to disrupted lipolysis in HCC cells by augmenting miR‐124‐3p leading to promoted cancer progression (Liu *et al*., 2018b).

### 3.4 Ovarian cancer

In ovarian cancer (OC), NEAT1 levels have been reported being upregulated in tissue of OC patients as well as in OC cell lines (An *et al*., 2017; Chen *et al*., 2016; Ding *et al*., 2017; Liu *et al*., 2018c). High NEAT1 levels correlate with tumor grade, occurrence of metastasis, and an unfavorable prognosis and could therefore be a potential biomarker for OC (Chen *et al*., 2016). Ding *et al*. showed that NEAT1 overexpression increases OC cell proliferation and decreases apoptosis by negatively regulating miR‐34a‐5p which in turn loses its repressor function on the antiapoptotic oncogene B‐cell lymphoma‐2 (BCL‐2; Tsujimoto *et al*., 1985) consequently leading to cancer cell proliferation (Ding *et al*., 2017). Another study shed more light on paclitaxel‐resistant OC by showing that NEAT1 sponges miR‐194 resulting in an upregulation of the EMT‐associated transcription factor zinc finger E‐box‐binding homeobox 1 (ZEB1; Zhang *et al*., 2015) leading to chemoresistance of OC cells (An *et al*., 2017). In the recent work of Liu *et al*., repression of NEAT1 was proven to inhibit the metastasis‐related gene Rho‐associated coiled‐coil containing protein kinase 1 (ROCK1). NEAT1 and ROCK1 were identified as being targets of the tumor‐suppressive miR‐382‐3p. NEAT1 upregulation is promoting OC progression and metastasis by regulating the ROCK1/miR‐382‐3p axis, or more precisely, NEAT1 promotes ROCK‐mediated formation of metastasis by acting as a ceRNA for miR‐382‐3p in OC (Liu *et al*., 2018c).

### 3.5 Prostate cancer

An interaction of NEAT1 with steroid receptor coactivator‐3 (SCR‐3), which is essentially needed for prostate cancer cell proliferation and growth (Zhou *et al*., 2005) via promoting insulin‐like growth factor receptor 1 (IGFR1) transcription followed by AKT signaling activation in prostate cancer, was observed by Xiong *et al*. Subsequently, the group was able to prove that NEAT1 increases AKT phosphorylation via the IGFR1 pathway promoting cancer cell growth (Xiong *et al*., 2018). An increased occurrence of mutations in the NEAT1 promoter was recently reported in patients who underwent androgen deprivation therapy in castration‐resistant prostate cancer (Wedge *et al*., 2018) highlighting the correlation between NEAT1 upregulation and resistance to androgen receptor antagonists (Chakravarty *et al*., 2014). A recent study of Li *et al*. showed that cell division cycle 5‐like protein (CDC5L) is a regulatory target of NEAT1 and that knockdown of NEAT1 results in reduced expression of AGRN, which is a direct target of CDC5L and a interaction partner of transforming growth factor beta 1 (TGFβ1) leading to DNA damage, cell cycle arrest ultimately resulting in decreased cancer cell growth and tumorigenesis in prostate cancer cells (Li *et al*., 2018b).

### 3.6 Nasopharyngeal carcinoma

Recent research could draw connections between NEAT1 and nasopharyngeal carcinoma (NPC). Also, in NPC NEAT1 is differentially expressed and its expression influences cancer cell characteristics. Liu *et al*. and Chen *et al*. report an upregulation of NEAT1 in cancer tissue and NPC cell lines and showed that knockdown of this lncRNA leads to inhibited proliferation, chemoresistance, and induced apoptosis (Cheng and Guo, 2017; Liu *et al*., 2018a). Increased NEAT1 levels found in NPC tissue and cells negatively correlated with expression levels of the tumor‐suppressive (Sampson *et al*., 2007) miRNA let‐7a‐5p resulting in upregulation of the oncogenic (Hrustanovic and Bivona, 2016) Ras‐MAPK‐signaling pathway; thus, authors conclude a contribution of the NEAT1/let‐7a‐5p axis to chemoresistance in NPC by modulation of Ras‐MAPK signaling (Liu *et al*., 2018a). Chen *et al*. proved a direct interaction and negative correlation of NEAT1 with miR‐124, a known tumor‐suppressive miRNA (Feng *et al*., 2015). miR‐124 regulates proliferation and apoptosis via NF‐κB—known to be activated during NPC progression (Sun *et al*., 2012)—or more precisely, upregulation of NEAT1 results in reduced miR‐124 expression leading to increased NF‐κB signaling in NPC (Cheng and Guo, 2017). Contradictory findings are presented in the work of Wang *et al*. where they showed (a) a significant downregulation of NEAT1 in NPC patient tissue, (b) that high expression levels are associated with a better survival in patients, and (c) that NEAT1 knockdown boosted migration but had no effect on proliferation (Wang *et al*., 2017b). Maybe, these contradictory findings are the result of the often very little sample sizes (e.g., microarray data control (*n* = 3) vs cancer tissue (*n* = 25)) or the different technical approach. The other groups had significant proliferation changes and proved these data by rescue experiments (Cheng and Guo, 2017; Liu *et al*., 2018a).

### 3.7 NEAT1 in other cancer types

**Figure 1:**
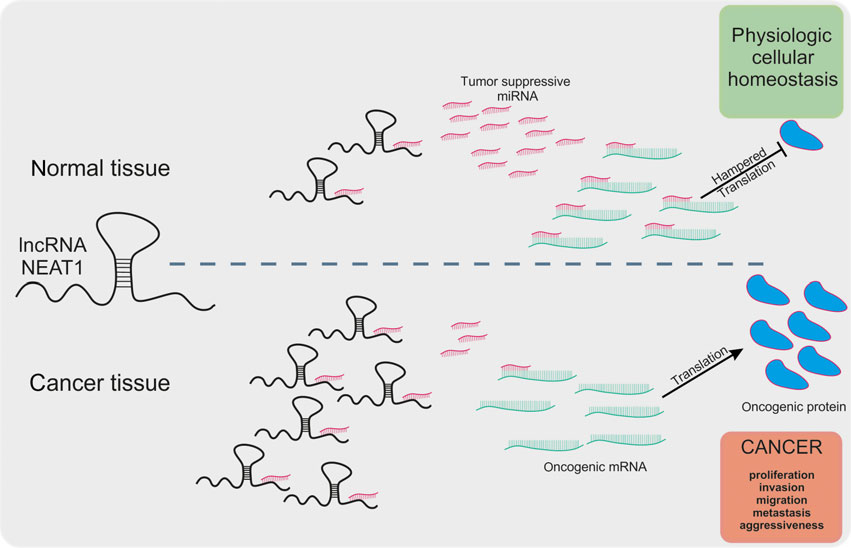
Schematic representation of the consequences of elevated NEAT1 expression levels in the context of cancer. (Upper panel) In normal tissue, NEAT1 expression levels are low; therefore, tumor‐suppressive miRNA are not sponged which enables them binding to oncogenic miRNA resulting in a hampered translation and low levels of oncogenic proteins. (Lower panel) In cancer tissue and cancer cell lines NEAT1 expression levels are high. Tumor‐suppressive miRNA are sponged by NEAT1 resulting in reduced binding of these miRNA to oncogenic mRNA. High numbers of these mRNA are translated to oncogenic proteins and cancer cell proliferation, invasion, migration, etc. are promoted.

A contribution of NEAT1 to cancer progression has recently been investigated in the following cancer types as well, that is, gastric cancer (Tan *et al*., 2018), osteosarcoma (Hu *et al*., 2018; Wang *et al*., 2017a), glioblastoma (Gong *et al*., 2016; Yang *et al*., 2017b), oral and esophageal carcinoma (Huang *et al*., 2018; Li *et al*., 2017a), clear cell renal carcinoma (Liu *et al*., 2017), and cervical carcinoma (Han *et al*., 2018; Wang and Zhu, 2018). Although these cancer entities are completely different regarding their localization, progression, molecular, and cellular features, they have one thing in common, that is, the NEAT1 phenotype namely (a) highly increased NEAT1 levels in tumor tissue and cancer cell lines. In all these different cancer types, upregulation of NEAT1 is associated with tumor stage and progression, metastasis, and an unfavorable patient survival. (b) Knockdown of NEAT1 results in a reduction of cancer cell growth, proliferation, migration, invasion, and an increase in apoptosis *in vitro* as well as in reduced tumor size and metastasis *in vivo*. In most cases, NEAT1 acts as ceRNA for a specific miRNA, therefore reducing the expression levels of the respective miRNA and consequently leading to the modulation—that is, mostly upregulation—of known oncogenic proteins (Fig. 1). More precisely, the underlying cellular mechanism is comparable in the above‐mentioned cancer types, though they only differ in the respective miRNA and modulated oncogenes, which are listed in Table 1.

**Table 1:** Interplay of certain miRNA with NEAT1 in diverse cancer types and the corresponding modulated protein targets (arrows indicate upregulation (↑) or downregulation (↓) of the respective factor). Ca, carcinoma; PBS, predicted binding site; Luc, Luciferase promotor assay; PD, RNA pull‐down assay; bis‐sequ., bisulfite sequencing

Cancer type	miRNA	3′–5′ sequence	PBS to NEAT1	PBS investigated in literature	Experimental method	Target protein	References
Nonsmall lung cancer	181a‐5p	UGAGUGGCUGUCGCAACUUACAA	4	1066	Luc, PD	↑HMBG2	Li *et al*. (2018a)
	377‐3p	UGUUUUCAACGGAAACACACUA	5	No details	Luc	↑E2F3	Zhang *et al*. (2017a)
	98‐5p	UUGUUAUGUUGAAUGAUGGAGU	3	4179^a^	Luc, PD	↑MAPK6	Wu *et al*. (2017)
Breast cancer	101‐3p	AAGUCAAUAGUGUCAUGACAU	1	12 605	Luc	↑EZH2	Qian *et al*. (2017)
	211‐5p	UCCGCUUCCUACUGUUUCCCUU	3	3209	Luc	↑HMGA2	Li *et al*. (2017d)
	488	UACCCUGUAGGAUGUAUACGUU	2	2331^a^	Luc	↑ZEB1	Jiang *et al*. (2018b)
	548ar‐3p	CGUUUUUAUUGACGUCAAAAU	0	2443 RNA hybrid	qPCR		Ke *et al*. (2016)
	129‐5p	CGUUCGGGUCUGGCGUUUUUC	3	No details	qPCR, bis‐sequ.	↑WNT4	Lo *et al*. (2016b)
Hepatocellular Ca	129‐5p	CGUUCGGGUCUGGCGUUUUUC	3	10 197	PD	↑VCP, ↓κB	Fu *et al*. (2017)
	613	CCGUUUCUUCCUUGUAAGGA	5	1863^a^	Luc	↑DCLK1	Wang *et al*. (2016, 2017c)
	485‐5p	CUUAAGUAGUGCCGGUCGGAGA	5	4456	Luc	↑STAT3	Zhang *et al*. (2018)
	139‐5p	UGACCUCUGUGCACGUGACAUCU	2	1588^a^	Luc, PD	↑TGFβ1	Tu *et al*. (2018)
	124‐3p	AACCGUAAGUGGCGCACGGAAU	3	2928	Luc	↑ATGL	Liu *et al*. (2018b)
Ovarian Cancer	34a‐5p	UGUUGGUCGAUUCUGUGACGGU	5	14 939	Luc	↑BCL2	Ding *et al*. (2017)
	194‐5p	AGGUGUACCUCAACGACAAUGU	2	3639	Luc	↑ZEB1	An *et al*. (2017)
	382‐3p	UUCACAACAGGCACUUACUAA	3	22 189^a^	Luc	↑ROCK	Liu *et al*. (2018c)
Gastric Cancer	506‐3p	AGAUGAGUCUUCCCACGGAAU	3	2928	Luc, PD	↑STAT3	Tan *et al*. (2018)
Cervical Ca	193b‐3p	UCGCCCUGAAACUCCCGGUCAA	3	1991	Luc, PD	↑Cyclin D1	Han *et al*. (2018)
	101	AAGUCAAUAGUGUCAUGACAU	1	12 605	Luc	↑FOS	Wang and Zhu (2018)
Nasopharyngeal Ca	let‐7a‐5p	UUGAUAUGUUGGAUGAUGGAGU	3	14 917	Luc	↑Ras‐MAPK	Liu *et al*. (2018a)
	124‐3p	AACCGUAAGUGGCGCACGGAAU	3	3252	Luc, PD	↑NFκB	Cheng and Guo (2017)
Oral squamous cell Ca	129‐5p	CGUUCGGGUCUGGCGUUUUUC	3	‐	Luc	↑CTBP2	Li *et al*. (2017a)
	365‐3p	UAUUCCUAAAAAUCCCCGUAAU	3	1901	Luc	↑RGS20	Huang *et al*. (2018)
Clear cell renal Ca	34a‐5p	UGUUGGUCGAUUCUGUGACGGU	5	14 939	Luc	↑c‐MET	Liu *et al*. (2017)
Osteosarcoma	34c‐5p	CGUUAGUCGAUUGAUGUGACGGA	5	14 938	qPCR	↑BCL2+ ↑CCND1	Hu *et al*. (2018)
	194‐5p	AGGUGUACCUCAACGACAAUGU	2	3639	Luc		Wang *et al*. (2017a)
Glioblastoma	107	ACUAUCGGGACAUGUUACGACGA	1	1514	Prediction, functional assays	↑CDK6	Yang *et al*. (2017b)
	let‐7e‐5p	UUGAUAUGUUGGAGGAUGGAGU	3	14 917,14 737	Luc	↑NRAS	Gong *et al*. (2016)

a Investigated miRNA binding site was not predicted with Starbase database.

## 4 Conclusion NEAT1 in cancer

In most cancer types, NEAT1 seems to be upregulated in cancer tissue compared to the corresponding noncancerous tissue as well as in the investigated cancer cell lines. High levels of NEAT1 have been shown to be associated with advanced tumor stage and cancer progression, the occurrence of metastasis, and poor patient survival. Knockdown of this lncRNA is associated with inhibition of proliferation, migration, invasion, increased apoptosis as well as decreased tumor size, and fewer metastases. This review highlights the function of NEAT1 as competitive endogenous RNA which is sponging many different miRNA in cancer and consequently leading to the modulation of oncogenic factors driving cancer related processes such as proliferation, invasion, migration, and often promoting epithelial to mesenchymal transition.

Although the NEAT1‐ceRNA hypothesis seems to be of common acceptance, there is one contradiction when regarding the underlying mechanism of miRNA processing, that is, miRNA are transcribed, modified, and processed into hairpin‐shaped pre‐miRNA in the nucleus followed by transport into the cytoplasm via Exportin‐5/Ran‐GTP complex where the hairpin is cut and the mature miRNA strand is incorporated into the RNA‐induced silencing complex (RISC) now able to interact with its target mRNA (Shukla *et al*., 2011). Based on this fundamental principle, miRNA sponging is supposed to happen in the cytoplasm. This is contradictory to the consistently described nuclear localization of NEAT1 (Clemson *et al*., 2009; Mito *et al*., 2016; West *et al*., 2016; Yamazaki *et al*., 2018a). As depicted in Table 1, miRNA binding sites on NEAT1 are mostly in the long isoform, strictly locating in nuclear paraspeckles (Nakagawa *et al*., 2011). Most reports demonstrating ceRNA function of NEAT1 have performed luciferase reporter assays showing a NEAT1‐dependent expression regulation of the respective miRNA or even pull‐down assays proving a direct interaction of NEAT1 with the investigated miRNA (Table 1). In our opinion, there are two possibilities allowing NEAT1 to act as ceRNA (a) NEAT1 is transported into the cytoplasm or (b) miRNA are transported in the nucleus. (a) Several reports demonstrate that lncRNA translocate from the nucleus to the cytoplasm upon cellular stress. In case of the lncRNA FILNC1, it is post‐transcriptionally methylated upon stress induction and subsequently exported into the cytoplasm (Xiao *et al*., 2017). Stress‐induced lncRNA get 5′‐capped, escape nuclear degradation, and are exported into the cytoplasm (Galipon *et al*., 2013). Therefore, it could be possible that NEAT1 undergoes post‐transcriptional modification and/or 5′‐capping, and gets exported to the cytoplasm, therefore possibly escaping conventional detection methods. The study of Nishizawa *et al*. is ruling out this possibility since they show that LINC00152 but not NEAT1 is relocating to the cytoplasm upon cellular stress (Nishizawa *et al*., 2018). (b) Upon cellular stress, miRNA, siRNA, and oligonucleotides are transported in the nucleus via a stress‐induced response complex (SIRC). Castanotto *et al*. showed that MALAT1 (also known as NEAT2 which was found in parallel to NEAT1 (Hutchinson *et al*., 2007) and is—as NEAT1—strictly localizing to the nucleus (Ip and Nakagawa, 2012)) is degraded in the nucleus because miR‐9 is transported there and directly targets MALAT1. Authors propose that the process of stress‐induced miRNA relocation to the nucleus is a universal process (Castanotto *et al*., 2018) enabling ceRNA function of NEAT1 in theory. If this is the way of ceRNA function of NEAT1 or if there is another explanation of the observed processes, then it still needs to be further investigated, but there are too many reports denying a role of NEAT1 in cancer.

The amount and diversity of involved miRNA, oncogenic factors, and pathways even within one cancer type underline the complexity of this malignant disease. The central molecular role of NEAT1 is an important shared feature in all cancers and indicates to the enormous potential of NEAT1 as a target in cancer therapy. As many authors conclude high levels of NEAT1 in cancer patients could serve as a useful prognostic biomarker. Although extensive research has so far shed light on the implication of NEAT1 in cancer biology and predicts promising potential for the use of this lncRNA in cancer diagnosis, prognosis, and therapy, more studies are needed toward therapeutic interventions.

## Author contributions

CK and MP designed the structure of the review; CK and FP performed literature research; CK, FP, and MP wrote the paper.

## Conflicts of interest

The authors have no conflicts of interest to declare.
